# C-754-04. GM-CSF Ameliorates Inflammatory Responses Caused by *Pseudomonas* in a Juvenile Mouse Model of Burn Injury

**DOI:** 10.1093/jbcr/irag033.032

**Published:** 2026-04-06

**Authors:** Julia A Penatzer, Ramya Chandran, Pranav Bodempudi, Rajan K Thakkar

**Affiliations:** Burn Center at Nationwide Children's Hospital, Columbus, OH; Nationwide Children's Hospital, Columbus, OH; Nationwide Children's Hospital, Columbus, OH; Nationwide Children's Hospital, Columbus, OH

## Abstract

**Introduction:**

Pediatric thermal injury, specifically burns with ≥20% total body surface area (TBSA), induces heightened inflammation and immune suppression, and are most at risk for adverse clinical outcomes (e.g., infections). Immunomodulating therapeutics, such as granulocyte macrophage colony-stimulating factor (GM-CSF), have been of interest to augment the immune response following thermal injury. Using a well-established juvenile mouse model of scald burn injury with bacterial infection we hypothesized GM-CSF delivered prior to infection will ameliorate inflammation and improve survival.

**Methods:**

Five-week-old BALB/c mice (n = 6-10/group) obtained a well-established full-thickness burn injury by exposing skin to a 98°C water bath for 10 seconds (~30% TBSA). Shams were exposed to room temperature water. Three days post-burn mice received either a single intraperitoneal injection of 1 μg GM-CSF or phosphate buffered saline. Mice randomly assigned to receive an infection were given a subcutaneous injection in the burn wound of 1 x 10^7 colony forming units (CFU) *Pseudomonas aeruginosa* (PA) on day 4 to simulate hospital acquired infections. Day seven post-burn injury, blood, lung, spleen, and burn tissue samples were collected. Spleens were analyzed by flow cytometry. Burn wound and lung samples were analyzed by immunohistochemistry. Plasma was analyzed for cytokine and soluble protein measurements by ELISAs. Viable burn tissue was analyzed by ELISA and RNA-sequencing to assess local inflammation. Values from the experimental groups were normalized to mean of the sham mice and analyzed by use of two-way analysis of variance, followed by Fisher’s LSD. A *p*-value <0.05 was used to indicate a significant difference between groups. Differentially expressed genes identified by RNA-sequencing were analyzed using QIAGEN's Ingenuity Pathway Analysis to identify network and cellular pathway interactions.

**Results:**

Burn injury with PA infection resulted in increased inflammation systemically and within the wound bed compared to burn alone, but treatment with a single dose of GM-CSF given prior to infection effectively ameliorated inflammation. PA increased systemic soluble B- and T-lymphocyte attenuator (BTLA) concentrations and decreased CD27, but BTLA was reduced in GM-CSF treated mice. Systemic leukocytes in the spleen and lungs were significantly reduced after infection compared to burn alone, but PA infected mice given GM-CSF had increased immune cells. Further, PA + GM-CSF mice indicated an increased wound healing network compared to infected mice who did not receive treatment.

**Conclusions:**

These findings provide early evidence that GM-CSF may represent a viable treatment option to improve clinical outcomes after pediatric burn injury.

**Applicability of Research to Practice:**

This data provides early pre-clinical evidence for the use of immunomodulators to augment the immune response following thermal injury to improve inflammation and prevent infection.

**Funding for the study:**

NIH - NIGMS.

